# Characteristics of parthenogenesis in *Cacopsylla ledi* (Flor, 1861) (Hemiptera, Sternorryncha, Psylloidea): cytological and molecular approaches

**DOI:** 10.3897/CompCytogen.v11i4.21362

**Published:** 2017-11-24

**Authors:** Seppo Nokkala, Valentina G. Kuznetsova, Christina Nokkala

**Affiliations:** 1 Laboratory of Genetics, Department of Biology, University of Turku, FI-20014, Turku, Finland; 2 Department of Karyosystematics, Zoological Institute, Russian Academy of Sciences, St. Petersburg 199034, Russia

**Keywords:** *Cacopsylla ledi*, Psylloidea, apomictic parthenogenesis, triploid females, diploid females, rare males, *COI* haplotypes

## Abstract

Characteristics of parthenogenesis in *Cacopsylla ledi* (Flor, 1861) were analyzed using cytological and molecular approaches. In all three populations studied from Finland, i.e. Turku, Kustavi and Siikajoki, males were present at a low frequency but were absent from a population from Vorkuta, Russia. In a follow-up study conducted in the Turku population during 2010–2016, the initial frequency of males was ca. 10 % and showed no intraseasonal variation, but then dramatically decreased down to approximately 1–2 % level in seasons 2015–2016. Male meiosis was chiasmate with some traces of chromosomal fragmentation and subsequent fusions. In most females, metaphase in mature eggs included 39 univalent chromosomes which indicated apomictic triploidy. Only a small fraction of females was diploid with 13 chiasmate bivalents. The frequency of diploid females approximately equaled that of males. *COI* barcode analyses showed that triploid females (N = 57) and diploids (7 females and 5 males) displayed different haplotypes, demonstrating that triploid females reproduced via obligate parthenogenesis. The rarity of diploids, along with the lack of males’ preference towards diploid females, suggested that most likely diploids were produced by rare triploid females which shared the same haplotype with the diploids (not found in the present analysis). Minimum haplotype diversity was detected in the Turku population, but it was much higher in Vorkuta with some indication for the mixed origin of the population. We suggest that functional diploids produced in a parthenogenetic population can give rise either to a new parthenogenetic lineage or even to a new bisexual species.

## Introduction

The great majority of psyllid species are characterized by bisexual reproduction. However, some members of, at least, in two genera, *Cacopsylla* Ossiannilsson, 1970 and *Trioza* Foerster, 1848 include all-female populations and are, therefore, suggested to be parthenogenetic. These are *C. ledi* Flor, 1961, *C. rara* (Tuthill, 1944), *C. myrtilli* W. Wagner, 1947, C. myrtilli ssp. canadensis Hodkinson, 1978, *T. pletschi* Tuthill, 1944 and *T. abdominalis* Flor, 1861 (Ossiannilsson 1972, Hodkinson 1976, 1978, Gegechkori 1985). Parthenogenesis of this kind is called thelytoky which is characterized by the presence of females that produce only daughters without fertilization. Thelytoky can be obligatory if only parthenogenetic populations are present throughout the whole distribution range of a particular species. Nevertheless, it can be facultative if both bisexual and parthenogenetic reproduction occurs, with cyclical parthenogenesis in aphids as a well-known example (Normark 2003, Vershinina and Kuznetsova 2016).

However, identifying facultative thelytokous taxa is usually difficult. In many animal taxa the existence of so called rare males was reported for some parthenogenetic lineages (Blackman 1972, Palmer and Norton 1990, Butlin et al. 1998, Martens 1998, Rispe et al. 1999, Simon et al. 1999, Delmotte et al. 2001, Snyder et al. 2006, Domes et al. 2007, Engelstadter et al. 2011, Maccari et al. 2013, Nokkala et al. 2013). These males can be either nonfunctional, i.e. incapable of producing haploid gametes (Taberley 1988, Nokkala et al. 2013) or functional with normal gamete production (reviewed by Maccari et al. 2013). Both kinds of males were found in geographically separate populations in the parthenogenetic psyllid *Cacopsylla myrtilli* (Nokkala et al. 2013). Females of this species are apomictic triploids (Nokkala et al. 2008). Cytological analysis has also discovered diploid females coexisting with the rare males especially at high altitude. These rare males, although being nonfunctional, copulate randomly with both parthenogenetic and “sexual” diploid females (Nokkala et al. 2015).

The characteristics of parthenogenesis in the Holarctic species *C. ledi* are poorly known. The species lives on wild rosemary *Ledum palustre* Linnaeus, 1753, and was previously recorded from Fennoscandia, the Baltic countries, Poland, Germany, Russia, Japan and Alaska (Ossiannilsson 1992). In addition to all-female populations, populations with males are known to exist, although quantitative frequencies of males are unknown. The males share the same chromosome number 2n = 25 (24 + X) with males of the genuine bisexual psyllid species (Kuznetsova et al. 1997).

In the present study, we planned to determine the frequency of males in four populations of *C. ledi*. In a follow-up study we planned to find out if the frequency of males would undergo any changes either during one reproductive season or between successive seasons. In addition, we planned to analyze chromosomes in mature eggs to determine the type of parthenogenesis and details of reproduction types of females in a particular population. Intrapopulational relationships between the females and males in a population were analyzed by using DNA barcode sequences.

## Material and methods

### Samples

Specimens of *C. ledi* were collected on *Ledum palustre* in four geographically separate locations, Turku, Kustavi and Siikajoki in Finland and Vorkuta in Russia (Table 1). Since this study was focused on the Turku population, samples were collected there during 2010–2016.

**Table 1.** Locations, number of females and males, male percentages and collection dates of *C. ledi* populations.

Location		Females	Males	Male percentage	Date
Kustavi	60°39'20"N, 21°18'12"E	310	4	1,3 %	1.8.2010
Siikajoki	64°39'32"N, 25°19'33"E	152	3	1,5 %	19.8.2010
Turku	60°29'56"N, 22°15'55"E	no adults			22.6.2010
		112	14	11,0 %	30.6.2010
		149	14	8,5 %	6.7.2010
		132	17	11,4 %	16.7.2010
		182	17	8,5 %	26.7.2010
		82	10	10,3 %	3.8.2010
		72	10	12,2 %	11.8.2010
		40	0	0,0 %	26.8.2011
		124	14	10,1 %	19.7.2012
		170	2	1,1 %	25.8.2015
		78	2	2,5 %	27.7.2016
Vorkuta, Russia	67°30'N, 64°02'E	10†	0	0,0 %	6.8.2013

†species confirmed by *COI* sequence among 39 individuals in the sample.

### Cytological study

Both female and male specimens of *C. ledi* were collected in June, July and early August to study spermatogenesis and sex ratios in certain populations. As females carried no mature eggs at that time, they were collected later in August for cytology (Table 1). Complete bodies of male individuals were put in 3:1 ﬁxative (96 % alcohol: glacial acetic acid) or stored in 96 % alcohol in the ﬁeld immediately after collection. To allow both chromosomal and haplotype analyses of the same individuals, collected living females were taken to the laboratory and dissected individually. For every female, the abdomen was put into the ﬁxative while the head and thorax parts were stored in alcohol. Cytology was performed following Nokkala et al. (2008). In brief, the abdomen was transferred from the fixative into a drop of 50 % acetic acid. Three to four mature eggs were extracted from the ovaries, the presence or absence of sperm in the spermatheca was recorded, and the rest of the abdomen was removed. To remove chorion, the eggs were cut in two parts with well sharpened tungsten needles. When yolk became transparent the eggs were squashed. The cover slips were removed by the dry ice method and slides were then immersed in fresh 3:1 fixative for 30 min and air dried. Air-dried slides were stained first with Schiff’s reagent for 30 min and then with 5% Giemsa for 40 min. Chromosomes were photographed with a BU4-500C CCD camera (BestScope International Limited, Beijing, China) attached to Olympus BX51 microscope (Olympus, Japan) using ISCapture Software version 2.6 (Xintu Photonics Co LTD, Xintu, China). Photographs were processed with Corel Photo-Paint X5 software.

### DNA barcoding

Total genomic DNA was extracted with DNeasy Blood and Tissue Kit (Qiagen) from complete bodies or thorax parts of adults. In cases when yield was below 20 ng / µl, the extractions were concentrated by precipitation with sodium acetate according to the standard procedure and the precipitate was solubilized in distilled water in one-fourth of the original elution volume. A fragment of *cytochrome c oxidase subunit I* (*COI*) gene was ampliﬁed using Applied Biosystems 2720 Thermal Cycler. Initially, the *C. myrtilli* speciﬁc primers HybCamyCO (forward) and HybymaCCO (reverse) were used and PCR reactions were carried out as described by Nokkala et al. (2015). Later on, to check the sequence at 5’ and 3’ ends, flanking primers HybCacoCO 5’-T7Promoter(F)-CTAACCATAARACTATTGGAAC-3’ (specifically designed forward primer) and a modified HCO (Folmer et al. 1994) reverse primer HybHCOMod 5’-T3-TAAACTTCAGGGTGACAAAAAATCA-3’ were used. PCR products were purified with QIAquick PCR Purfication Kit (Qiagen) and sequenced by Macrogen Europe (Amsterdam, the Netherlands). The sequences were trimmed to span a 638 bp stretch of the gene to match the available sequences of related *C. myrtilli* (Nokkala et al. 2015). Sequences obtained during this study have been deposited in GenBank under the accession numbers MF978762-MF978766.

## Results

### Population structure

The majority of individuals in all populations were females, while the frequency of males varied in different samples from 0 % to 12.2 % (Table 1). In Kustavi and Siikajoki, the frequencies were low, 1.3 % and 1.5 % respectively. Males were absent in the sample from *Ledum* in Vorkuta. In the Turku population, the frequency of males was higher, around 10 %, and showed no seasonal variation between 30.6–11.8 in 2010. The frequency remained high in 2012, but decreased dramatically to 1.1 % in 2015 and remained low in 2016. Low frequencies of males suggest that *C. ledi* reproduces parthenogenetically, and males represent the so-called rare males commonly found in parthenogenetic lineages.

### Cytology

**Figures 1–3.**
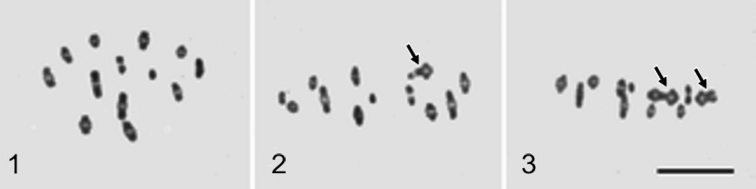
Male meiosis in *C. ledi*. **1** Metaphase I with normal karyotype, n = 12 + X (0) (from Kuznetsova et al. 1997) **2** Metaphase I. Arrow points to trivalent which is heterozygous for a fusion **3** Metaphase I. Arrows point to two trivalents which are heterozygous for fusions. Scale bar: 10 µm.

In *C. ledi* males, testes have four testicular follicles, in contrast to two, which is the common number in psyllids (Kuznetsova et al. 2012). The male karyotype was earlier reported to be 2n = 25 (24+X) by Kuznetsova et al. (1997), which was confirmed by the present study (Fig. 1). In male meiosis chiasma formation is normal; hence twelve bivalents and a univalent X chromosome are seen at metaphase I. However, males heterozygous for fusions are not uncommon (Figs 2 and 3); they can carry either a single heterozygous trivalent (Fig. 2) or two such trivalents (Fig. 3) Nevertheless, these metaphases originate from different testes of the same individual, thus indicating that the chromosome breaks leading to subsequent fusions have occurred in the germ line.

### Females

The chromosome number of females is most easily determined at metaphase I in mature eggs. For a closer study of the biology of females during one season, several samples were taken from the Turku population in 2010 starting on 30.6., when adults just appeared in the population (Table 1). First females with mature eggs were found not earlier than 11.8, when single mature eggs were found in very few females (Table 2). On 25.8.2015 all females carried several mature eggs in their ovaries. The egg-lying of females was studied in the laboratory using material collected in 2015. We found that females preferred to deposit their eggs on the underside of the uppermost narrow leaves of *Ledum* plants. This clearly indicates that *C. ledi* hibernates as an egg.

**Figures 4–5.**
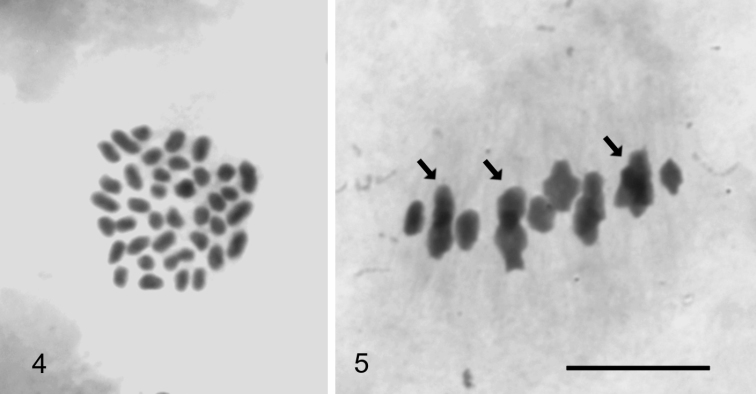
Female meiosis in *C. ledi*. **4** Metaphase from mature egg with 39 univalent chromosomes **5** Metaphase I plate with 13 chiasmate bivalents (12 + XX), sex chromosome bivalent cannot be identified. Arrows point to three overlapping pairs of bivalents. Scale bar: 10 µm.

Cytological analysis revealed that there were two kinds of females in the population. Mature eggs of the great majority of females showed 39 univalent chromosomes at metaphase, indicating that these females were apomictic triploids (Fig. 4). There were also a small number of females with a haploid number of chiasmate bivalents, i.e. those females were diploid (Fig. 5). Typically, there was only a single terminal or subterminal chiasma per bivalent. The frequency of diploid females within a particular sample varied from 30.0 % to 2.7 %, as shown in Table 2. It is noteworthy that the approximate tenfold drop-down in the frequency of diploid females between 2012 and 2015 is similar to the decrease found in male frequencies from 10.1 % to 1,1 % (Table 1). Unfortunately, no mature eggs were found in females collected in Vorkuta.

**Table 2.** Number of triploid and diploid females in Turku population.

Triploid females	Diploid females	Percentage of diploids	Collection date
2	1	N/A	11.8.2010
14	6	30,0 %	28.6.2011
36	1	2,7 %	25.8.2015
27	1	3,6 %	21.8.2016

### The absence of male mating preference

With both diploid and triploid females present in a population, it is interesting to find out whether males prefer to mate with diploid ones or, at least, can distinguish between these two. For this purpose, while making cytological preparations from females, spermathecae were also checked for the presence of sperm. However, only seven out of 71 females checked carried sperm in their spermathecae, two of them being diploids and the remaining five triploids (Table 3). This indicates that males are quite inactive in mating in general but occasionally mate either with diploids or triploids. Since the males do not discriminate against triploid females successful independent bisexual reproduction in *C. ledi* seems highly unlikely.

**Table 3.** Number of diploid and triploid females with sperm in their spermathecae.

Number of females checked					
triploid		diploid		Σ	collection date
sperm	no sperm	sperm	no sperm		
2		1		3	11.8.2010
1	9	1	3	14	26.8.2011
1	34		1	36	25.8.2015
1	16		1	18	21.8.2016
5	59	2	5	71	totals

### *COI* barcoding

DNA was isolated from alcohol preserved thorax parts of the cytologically studied individuals. A *COI* fragment of 638 nucleotides was sequenced from 57 triploid parthenogenetic females and 12 diploids (7 females and 5 males) from the Turku population. All triploids shared the same haplotype which was different from that of the diploids. These haplotypes differed by a particular transversion at the position 192, T (Turku 1 haplotype, MF978762) in triploids and A (Turku 2 haplotype, MF978763) in diploids. These data, therefore, demonstrated that triploid parthenogenetic from Turku produced exclusively triploid offspring. The haplotype diversity in the Turku population was low, but was much higher in Vorkuta. Turku 1 haplotype was also found in Vorkuta (2/11 females). In addition, three specific haplotypes from Vorkuta were found, Vorkuta 1 (1/11 females), Vorkuta 2 (5/11 females) and Vorkuta 3 (3/11 female) differing from Turku 1 by either a single nucleotide (Vorkuta 1 and 2) or three nucleotides (Vorkuta 3) (MF978764-MF978766).

## Discussion

Our findings show that details of parthenogenesis in *C. ledi* are similar to those found previously in *C. myrtilli* (Nokkala et al. 2015). Parthenogenetic females of *C. ledi* are triploid showing apomictic oogenesis in which normal meiosis is replaced by a modified mitosis. In addition to triploid females, there were also diploids showing normal chiasmate meiosis. Moreover, the presence of diploid females among obligate triploid parthenogenetic females was discovered for the first time in populations of *C. myrtilli* collected at various altitudes on the hill Rindhovda in southern Norway (Nokkala et al. 2015). Diploid females showing conventional meiosis were found at frequencies similar to those of rare males at three different altitudes. The highest percentage of both diploid females and males, 10%, was found in the high altitude (1000 m) population, above the tree line. Comparison of frequencies of diploids at different altitudes showed that environmental factors, like altitude, seem to significantly affect the production of diploids (Nokkala et al. 2015). The findings that rare males in those populations were nonfunctional, producing only diploid sperm (Nokkala et al. 2013), on one hand, and the fact that diploids showed the same *COI* haplotype as triploid females, on the other hand, collectively suggested that diploids resulted from reversions from triploidy and that the frequency of these reversions seemed to be influenced by environmental factors, e.g. altitude. In addition, reversions resulted in both males and diploid females with a similar frequency (Nokkala et al. 2015).

Haplotype diversity in the Turku population of *C. ledi* was extremely low, where the two recorded haplotypes differed by a single transition. However, much higher diversity was found in the Vorkuta population showing four different haplotypes among the small number of females studied. The close similarity of haplotypes Vorkuta 1 and Vorkuta 2 to that of Turku 1 suggests common ancestry. In contrast, Vorkuta 3 haplotype differed from all other haplotypes found in the population by three nucleotide changes, one of these being a transversion, indicating different origin of this haplotype.

In *C. ledi* triploid females and diploid females and males displayed different *COI* haplotypes. Our results prove that triploid females reproduce via obligate thelytoky. Those females, therefore, do not produce diploid females or males. To account for the occurrence of diploid individuals, it is tempting to speculate on the possibility of independent bisexual reproduction. Potentially, reproduction of this kind is possible, since diploid females display normal chiasmate meiosis and males despite some disturbances show virtually normal meiosis. Two observations, however, make bisexual reproduction unlikely. Firstly, the frequency of males in populations at Turku after the drop-down of diploids is very low and is the same magnitude or clearly below 10% like that of rare males in oribatid mites (Palmer and Norton 1990), in *Artemia* Leach, 1819 species (Maccari et al. 2013), or in *C. myrtilli* populations (Nokkala et al 2013). Secondly, those males do not discriminate against triploid females making bisexual reproduction highly unlikely. An alternative explanation implies that rare, still undiscovered triploid females which share the same haplotype with diploids are responsible for the production of diploid females and males via reversion from triploidy to diploidy. This means that the genotypes of triploid females would greatly affect their ability to produce diploids. In turn, this would also mean that *C. ledi* is an obligate parthenogenetic. However, additional studies are needed to confirm this suggestion.

Although in the short term it is difficult to see any advantage for the production of diploids in the long run they can provide further evolutionary opportunities for a parthenogenetic taxon. It is known that functional rare males can mate with closely related sexual females to give rise to a new parthenogenetic lineage. This type of parthenogenesis, known as contagious parthenogenesis (Simon et al. 2003), was analyzed in detail in *Daphnia pulex* Leydig, 1860 (Paland et al. 2005) and brine shrimps of the genus *Artemia* (Maccari et al. 2013). The origin of contagious parthenogenesis was also proven in the laboratory for *D. pulex* (Innes and Herbert 1988), for the aphid *Myzus persicae* (Sulzer, 1776) (Blackman 1972) and for *Artemia* spp. (Maccari et al. 2014). Apparently, new parthenogenetic lineages of contagious origin can also be produced by diploid females found in *C. myrtilli* (Nokkala et al. 2015) and in *C. ledi* (the present study), if these females are mated with males of a coexisting sexual species. The origin of contagious parthenogenesis could more easily occur via diploid females than through males as they are functional (or show normal meiosis) even in the case when males are nonfunctional (Nokkala et al. 2015). If both diploid females and functional rare males exist in a population, mating between diploids could occasionally result in diploid offspring, in spite of the fact that males do not discriminate against triploid females. Potentially, this could lead to the origin of a new bisexual species from a parthenogenetic lineage. This can also be the case in oribatid mites. Domes et al. (2007) considered phylogenetic relationships between certain taxa belonging to seven families of the oribatid mite group Desmonomata and concluded that a parthenogenetic lineage could evolve to a new parthenogenetic lineage or even to a new bisexual species. Apparently, the evolutionary potential of parthenogenetic lineages is high, complex and far more diverse than previously understood.

## Conclusion

Parthenogenetic females of *C. ledi* are triploid with apomictic meiosis. Triploid females reproduce via obligate parthenogenesis. We also suggest that rare males and diploid females, if present, are produced by triploid females by reversions from triploidy to diploidy. This probably demonstrates how a parthenogenetic taxon can give rise either to a new parthenogenetic lineage or even to a new bisexual species.
